# Systematic review of craniofacial osteosarcoma regarding different clinical, therapeutic and prognostic parameters

**DOI:** 10.3389/fonc.2023.1006622

**Published:** 2023-03-24

**Authors:** Verena Weber, Robert Stigler, Rainer Lutz, Marco Kesting, Manuel Weber

**Affiliations:** ^1^ Department of Operative and Restorative Dentistry, Medical University of Innsbruck, Innsbruck, Austria; ^2^ Department of Oral and Maxillofacial Surgery, University of Innsbruck, Innsbruck, Austria; ^3^ Department of Oral and Cranio-Maxillofacial Surgery, Friedrich-Alexander University Erlangen-Nürnberg (FAU), Erlangen, Germany

**Keywords:** craniofacial osteosarcoma, extracranial osteosarcoma, head and neck osteosarcoma, maxillofacial bone sarcoma, surgical therapy, chemotherapy, radiotherapy, survival

## Abstract

**Background:**

Osteosarcomas are the most common primary bone tumor while occurrence in the craniofacial skeleton is relatively rare. There are clinical differences of osteosarcomas regarding their location. In this regard craniofacial osteosarcomas (COS) have special characteristics. Extracranial osteosarcomas (EOS) occur mainly in the long bones of the extremities (tibia, humerus and femur). These tumors metastasize hematogenically at a very early stage. In comparison, COS are mainly localized in the mandible and maxilla, occur later in life and show significantly less and later metastasis and respond differently to adjuvant therapy. In the literature, clinical characteristics of COS and EOS are rarely compared directly. The aim of this systematic review is to answer the question whether COS and EOS exhibit fundamentally different clinical behavior and how they differ in terms of survival rates and response to different therapies.

**Methods:**

A systemic review was performed. Pubmed, Cochrane and Google Scholar were used as search engines. The literature research was done by using clearly defined terms and their links. 124 full texts were selected and evaluated for this review. The inclusion criteria were determined using the PICO model.

**Results:**

COS have significantly better survival rates, especially if they are located in the jawbone. Surgical R0 resection is crucial for therapeutic success. The study situation regarding the benefit of neoadjuvant chemotherapy in COS is very inhomogeneous. There is also no evidence for the benefit of adjuvant radio- or chemotherapy in COS. The large heterogeneity of the studies in terms of therapeutic concept, initial situation of the patients and outcome considered, as well as the small number of patients with craniofacial osteosarcoma were limiting factors.

**Conclusion:**

The results of this study show the clear therapeutic and prognostic differences between COS and EOS and underline the necessity to consider both types of osteosarcoma as independent tumor entities in future studies. Furthermore, the study highlights the importance of surgical R0 resection for the prognosis of COS patients. There is no evidence for therapeutic benefit of adjuvant/neoadjuvant radio-/chemotherapy in R0 resected COS cases.

## Introduction

Sarcomas are malignant neoplasms, which develop from cells of mesenchymal origin (1).

Osteosarcoma (OS) mainly affects teenagers and young adults (2). The age of manifestation is usually between 10 and 25 years (3). In this group of patients, the tumor occurs mainly in the long bones of the extremities, such as the tibia, humerus and femur (4).

According to the current classification of the *American Joint Committee on Cancer* (AJCC), osteosarcomas of the tubular bones are histologically divided into “high grade” and “low grade” tumors and additionally, a stage classification (stage 1-4) is performed, which depends on the tumor size, histological grade and metastasis (5). 90% of the OS are central, primary high grade tumors (3). The exact etiology of osteosarcoma is unknown (3).

The current gold standard of therapy for tubular bone OS is neoadjuvant chemotherapy, followed by surgical resection of the primary tumor and adjuvant chemotherapy. Primarily as a result of chemotherapy, the 5-year survival rate of OS has improved to 60-70% (6).

Osteosarcomas of the facial skull, the craniofacial osteosarcomas (COS), differ from the osteosarcomas of the axial skeleton (extracranial osteosarcomas, EOS) in their different clinical and biological behavior (7). COS are a rare subgroup of osteosarcomas with a share of about 10%. Of the malignant head and neck tumors, osteosarcomas account for less than 1% of cases (8). Most COS develop in the upper and lower jaw (9). The manifestation age of the COS is 10-20 years later than that of the extracranial OS, i.e. in the third to fourth decade of life (10).

The clinical characteristic of COS is the very rare rate of metastasis, especially in the lungs. In contrast to extracranial osteosarcomas (EOS), the difficulty of surgical resection of COS is the achievement of a R0 situation. In many cases this is complicated by the proximity of the tumor to important anatomical structures (8). In addition, the demand for an aesthetically and functionally good result in the face makes it difficult to achieve resection margins far into the healthy area (10). Therefore, recurrence is a common problem of COS and the most common cause of an unfavorable course. Kämmerer et al. described local recurrence as the most frequent cause of death of COS (1). Since it is easier to reach tumor-free resection margins in the mandible, OS of the mandible show a better prognosis than those of the maxilla (10).

The role of chemo- or radiotherapy in COS is unclear. Chemotherapy alone has no benefit in COS. It is unclear whether adjuvant chemotherapy can have a positive effect after surgical resection (7). Although osteosarcoma have different histologically defined origins, there are no relevant prognostic differences (1).

There is evidence that COS differ from EOS regarding their clinical behavior and response to multimodal therapy. There is little literature available directly comparing the treatment and outcome of COS and EOS. The current systematic review will assess the following question according to the PICO criteria:

What is the 5-year survival rate of patients with COS vs. EOS and what influence on the survival rate have the different therapeutic interventions (surgery, chemotherapy, radiotherapy and combinations)?

## Materials and methods

### Data sources

**Figure 1:**
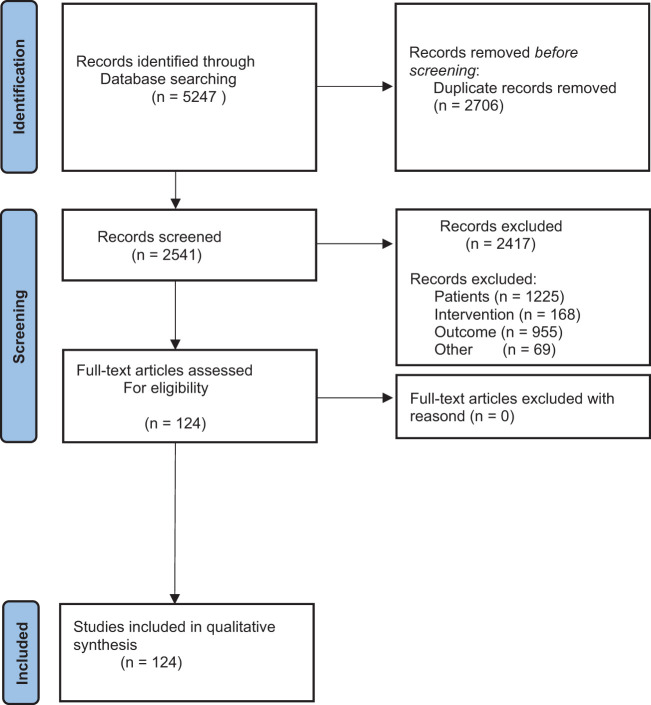
The figure shows the Prism Flow Diagram. It shows the systematic process of literature search.

This systematic review is conducted according to the Preferred Reporting Items for Systematic Reviews and Meta-Analysis (PRISMA) guidelines (see **Figure 1**) (11). The literature used consists of articles from professional journals as well as textbooks and dissertations. Pubmed, Cochrane and Google Scholar were used as search engines.

Literature in English and German, published from 2000 till 2021, was included in the search.

For the search terms “osteosarcoma” and its terms “therapy” and “survival rate” added with “AND”, studies were included in the results from 2010 onwards. Due to the low publication rate on craniofacial osteosarcoma, studies up to the year 2000 were included for the search terms “osteosarcoma of the head and neck”, “osteosarcoma of the jaw” and “craniofacial osteosarcoma”. Care was taken to include results from studies conducted worldwide. Review articles and original papers were included in the search (12).

Literature search was carried out using search strategies defined at the beginning of the work, which are shown in **Table S1**. First, the term “osteosarcoma” was used and combined with the term “therapy” using an AND. To include a wide range of possible therapies, these two terms were combined with “neoadjuvant chemotherapy”, “adjuvant chemotherapy”, “radiotherapy”, “surgical therapy” and “multimodal treatment”.

The next step was to link the generic term “osteosarcoma” with the term “survival rate”. The link again was made by AND and was concretized by the search terms: “stating”, “metastasis”, “local recurrence”, “tumor size” and “resection margins”.

In order to extend the search specifically to craniofacial osteosarcoma, the terms “osteosarcoma of the head and neck”, “osteosarcoma of the jaw” and “craniofacial osteosarcoma” were used (see **Table S1**). Two authors searched independently using the same keywords.

The papers found were transferred to the citation program *Endnote (Clarivate Analytics)* and examined using the PRISMA criteria.

### Criteria for inclusion and exclusion

Inclusion and exclusion of studies was performed according to the PICOS-criteria (13).

The main question of this systemic review was what is the 5-year survival rate of patients with COS vs. EOS and what influence on the survival rate do the different therapeutic interventions (surgery, chemotherapy, radiotherapy and combinations) show?

Complementary to the main PICOS-question this review should answer the following questions:

- What effect does the radicality of surgical resection (the resection margins) have on survival in craniofacial osteosarcomas?
- What role does the modality of therapy (surgery, chemotherapy, radiotherapy) play in survival rates?
- Is the gold standard of therapy for extracranial osteosarcomas also applicable to craniofacial osteosarcomas?
- How do extracranial and craniofacial osteosarcomas differ with respect to metastasis and local recurrence?

Only histologically confirmed OS were included in this analysis. Most of the papers have been sorted out because they deal with other tumor entities. Ewing sarcomas are considered in literature and were excluded in this analysis but also tumors like retinoblastomas, adenocarcinomas, liposarcomas or chondrosarcomas. Furthermore, animal studies were excluded. These were mainly studies on dogs and mice. Case reports were also excluded.

Studies focused on the “Outcome” survival rate were included in this review. Exclusion due to the category “Outcome” was performed when articles were focused on one specific aspect that was not relevant for this analysis like (e.g. special histologic parameters or molecular markers, radiologic parameters, functional outcome of surgery, compliance data, etc.). Regarding the PICOS criteria “Intervention”, studies describing patient collectives treated with surgery, chemotherapy and radiotherapy were included. Experimental treatments including immunotherapy targeted therapy, gene-therapy or viral therapy were excluded.

## Results

### Literature selection

A total of 5247 available primary studies were identified (see **Figure 1**). After applying the PRISMA diagram, duplicates were removed and the titles of the primary studies were screened (see **Table S1**). **Table S1** shows how many studies were included in the paper after screening titles, abstracts, and full texts. This information is given for all search terms used. **Table S2** shows the exclusion criteria based on the PICO model. It shows which studies were excluded based on the patient population, the intervention or the outcome. After reviewing the titles, the abstracts of 358 studies were read and evaluated. 233 studies contained relevant information for the present study. Full texts were available for 124 studies.

Percentages of 5-year survival were found in only 31 studies.

The study results are presented using several tables. The tables show the 5-year survival rates in COS (**Table 1**) and EOS (**Table 2**). Another table shows the survival rates related to the resection margins after surgical intervention (**Table 3**). Survival rates related to the different therapeutic interventions are shown in **Table 4**.

**Table 1:** 5-year survival of COS patients.

Author	Year	Localization	Number of patients	5-year survival	Declaration
Meazza C.	2014	Mandible/Maxilla	4	75%	-Only 4 Patients
Chen Y.	2017	Craniofacial (Maxilla/skull base 43.3 1%, Mandibula 57.56%)	157	50.96%	
Chen Y.	2016	Craniofacial (Maxilla/skull base 58 Mandibula 79)	137	Maxilla/skull base 56.90% Manibular 70.89%	-7 Patients with positiv margins, all of them died
Jasnau S.	2008	Mandible/Maxilla Extragnathic	49	88.1% 58.4%	-Median age 19.7 years -27% Osteosarcoma was secondary malignancy
König M.	2016	Craniofacial Mandibula/Maxilla 76% Extragentic 24%	42	44,7%	-50% of patients had prior malignancies and a familial predisposition -Only 36% R0
Fernandes R.	2007	Mandible/Maxilla	16	86%	
Eder-Czembirek C.	2019	Mandible/Maxilla	18	85.9%	
Jeong H.	2017	Mandible/Maxilla	26	73.5%	
Thariat J.	2013	Mandible	111	69.2%	-34 patients with R1, only 94.5% surgery
Granowski-LeCornu M.	2011	Mandible/Maxilla	Group 1 (treated 1967-1991): 30 Group 2 (treated 1992-2009): 17	-All Patients: 68% -Patients (1967-1991): 52% -Patients (1992-2009): 77%	-R0 only at 67.4% (83% R1 Maxillary)
Guo Z.	2017	Base of the skull	19	30.5%	-6 Patients R1

The table lists the 5-year survival rates of the considered studies of craniofacial osteosarcoma. The author of the study, the location of the included tumors and the 5-year survival rate are given. In addition, an explanation is added to show the specifics of each study.

**Table 2:** 5-year survival of EOS patients.

Author	Year/Study type	Localization	Number of patients	5-year survival	Declaration
Meazza C.	2014	Axial/Pelvic	16 8 pelvic 8 axial	25%	- 4 patients with p53 mutation - 25% showed metastases at diagnosis - not every patient was treated surgically/ for only 50% wide surgery was possible
Guillon M.	2011	Femur, tibia, humerus	15	55%	-40% showed metastases at diagnosis. -only 36% showed good histologic response to chemotherapy
Hagleitner M.	2011	Extracranial	102	53.5%	
Huang J.	2018	Proximal Tibia	69	87%	-inclusion criteria: no vessel/nerve involvement no metastases
Hung G.	2015	extremities	74 21.6% with metastases at diagnosis	77% 25% in patients with metastases 90.4% in patients without metastases	84.7% of patients show good response to chemotherapy
Wiromrat P.	2012	Extracranial	58	27.6% with amputation: 33.5% without amputation: 24.1%	25.9% of the patients were not treated 25.9% were treated only surgically and received an amputation 25.9% received amputation and chemotherapy (no info if neoadjuvant or adjuvant) 22.3% received chemotherapy only
Kamal A.	2016	Extracranial	132	14.6%	Patients did not receive multimodality therapy. Patients were treated only surgically either with limb salvage surgery or amputation

The table lists the 5-year survival rates of the considered studies of extracranial osteosarcoma. The author of the study, the location of the included tumors and the 5-year survival rate are given. In addition, an explanation is added to show the specifics of each study.

**Table 3:** Survival rate related to resection margins in COS patients.

Author	R0/R1	5 year OS	5 year DSS	5 year DFS
Jasnau S	R0 R1	88.6% 38.8%		
König M	R0 R1		66.7% 39.3%	
Chen Y	R0 R1		53.33% 0%	
Chen Y	R0 R1		68.46% 0%	
Krishnamurthy A	R0 R1			53.1% 0.00%
Baumhoer D	R0 R1		78.3% 21.8%	

The table shows the 5-year survival rate and 5 years disease-specific survival rates of the studies with respect to the resection margins. The author, the surgical resection margins (R0/R1) and the 5-year survival are given. OS, Overall-survival; DSS, Disease-specific-survival.

**Table 4:** Survival rate related to therapeutic intervention in COS patients.

Author	Year		Patients		Therapy	5 Year OS	1 Year DSS	2Year DSS	5 Year DSS
Baumhoer D.	2014		214 Patients with Osteosarcoma of the jaws (Median age 37 years)		Surgery+ neoadjuvant CT				50.1%
					No neoadjuvant CT				69.9%
					Surgery + adjuvant CT				47.3%
					No adjuvant CT				74.6%
					Surgery + neoadjuvant and adjuvant CT				44%
					No neoadjuvant and adjuvant CT				68.8%
Jasnau S.	2008		49 patients -Median age 19,7 years -27% Osteosarcoma was secondary malignancy		CT+ surgery	74.5%			
					Surgery	66.7%			
Mücke T.			-36 patients -only 12 patients received neoadjuvant CT		Surgery	41.7%			
					Neoadjuvant CT + surgery	66.7%			
Chen Y.	2017		157 Patients (91 Mandibula, (68 Maxilla)		Surgery	52.31%	76.92%	60.0%	52.31%
					Surgery + CT	46.67%	86.67%	60.0%	46.67%
					Surgery + CT+RT	47.89%	95.65%	78.26%	47.83%
					Surgery+RT	51.85%	81.48%	67.96%	51.85%

The table shows the impact of different therapeutic interventions on survival rates of patients with craniofacial osteosarcoma. The considered patient collective is described in more detail related to the number of patients and the respective characteristics of the collective.

DSS, disease specific survival.

The results of the tables are now described in detail.

### Survival rates in craniofacial osteosarcoma

The results of the literature search related to the 5-year survival rate in COS are shown in **Table 1**. **Table 1** reveals a wide range of survival rates. This can be explained by the very inhomogeneous patient population of the studies considered. Both the initial clinical situation and the applied therapy regimens vary considerably. However, it can be stated that all studies show the best survival rates with tumor localization in the mandible and with R0 resection.

The first study listed in **Table 1** (Meazza et al.) is the only study included in this review that directly compares survival data between COS and EOS. In terms of survival rates, the following results were shown: Patients with osteosarcoma of the jaw showed a 5-year survival rate of 75%. If the osteosarcoma was located in the pelvis or axially, the 5-year survival rate was 25% (see **Table 1**) (14).

The two studies listed in **Table 1** by Chen et al. both showed a patient population with approximately 50% tumor localization in the mandibula and 50% in the maxilla/skull base (see **Table 4**) (15, 16). The second study by Chen et al. (2016) listed in **Table 1** gives a survival rate of 56.9% for patients with tumor localization in the maxilla or skull base. Considering only the patients with tumor localization in the mandible, the 5-year survival rate was 70.89% (16). In a large-scale study by Lee et al., data from 541 patients with COS were analyzed. 55.6% of the patients showed tumor localization in the skull/facial bone and 44.4% of the patients had the mandible as localization. This study showed a longer median survival (10.4 years) of mandibular osteosarcoma compared to the rest of the skull/facial bone (6.3 years) (17). A study that proves the thesis of poorer survival rates with tumor localization outside the jaw bones is the last study listed in **Table 1**. Guo Z. et al. analyzed 19 patients with osteosarcoma of the skull base. The 5-year survival rate of these patients was only 30.5%. Guo et al. explained the low survival rate by a high recurrence rate (1-year recurrence rate 47.0%, 2-year recurrence rate 68.8%) and proximity to complex and anatomically important structures (18).

### Survival rates in extracranial osteosarcoma

In **Table 2**, all data, from the analyzed studies, providing information on the 5-year survival rate of patients with EOS were presented. Looking at the survival data of EOS also reveals a very broad spectrum. Most of the patients with EOS reported a 5-year survival rate of about 50%. Better data regarding survival could be shown in studies with special inclusion criteria of the patient collective. The best survival rates were seen in patients who had no vascular or nerve involvement of the tumor on imaging and no metastatic disease at diagnosis (19). Hung et al. compared the 5-year survival rates of patients with and without metastases at diagnosis. Patients without metastases showed a 5-year survival rate of 90.4%. Patients with metastases showed a 5-year survival rate of only 25% (19).

The presence of metastases and histologic response to chemotherapy correlated significantly with survival in EOS (19). 84.7% of the patient population in the Hung G. et al. study showed a good response (tumor necrosis rate of >90%) to neoadjuvant chemotherapy (19). Comparing the results with the results of Baumhoer et al. in this regard, who investigated the response of COS to chemotherapy, a significantly worse result is shown. The response of 8 patients was investigated. Of these, 6 were poor responders (>90% vital tumor remnants) (20).

Significantly worse survival data were seen when patients did not receive multimodality therapy consisting of neoadjuvant chemotherapy followed by large-scale resection and adjuvant chemotherapy (20). This is also confirmed by the data of the last two studies in **Table 2** by Wiromrat et al. and Kamal et al. They showed the survival rates of a patient population that did not receive multimodal therapy (21, 22). However, it should be noted that 25.9% of the patients in Wiromrat et al. did not receive any therapy. Wiromrat et al. justified this by the fact that many patients only presented at a very advanced stage and denied therapy (21). Furthermore, the localization of the tumor in the axial skeleton indicates a poor prognosis compared to the extremities (23).

### The impact of metastases on survival rates in COS and EOS

The studies considered in this review clearly demonstrated the negative impact of metastases on patient survival rates in both COS and EOS.

The importance of metastasis in COS is illustrated by the studies of Eder-Czembirek et al., Granowski-LeCornu et al. and Baumhoer et al. (20, 24, 25)

At the Medical University of Vienna, Eder-Czembirek et al. analyzed 18 patients with osteosarcoma of the jaw and compared the results of their study group with the results of the “DOESAK registry group (214 patients)”. Comparing the 5-year disease-specific survival rates, the study group of Eder-Czembirek et al. showed a 5-year disease-specific survival rate of 100% without metastases. If they developed metastases (22% of patients), the survival rate was 80%. The DOESAK group showed the same results: without metastases the 5-year survival rate was 78.3%. If metastases developed, the value dropped to 19.7% (24). Baumhoer et al. showed that patients with osteosarcomas of the jaw developed metastases significantly less frequently (17.6%) and later in the course (mean 26 months after diagnosis) compared to patients with EOS (20). This is another indicator that osteosarcomas of the jaw bones cannot be compared with osteosarcomas of the peripheral skeleton. Extracranial osteosarcomas must be considered systemic disease. Without a multimodal therapy concept, >90% of patients develop metastases (20). Metastasis had a negative impact on the 5-year survival rate of patients with osteosarcomas of the jaws (78.3% without metastases/19.7% with metastases) (20). Only 1.9% of patients of Baumhoer et al. already showed metastases at the time of diagnosis. In these patients, the disease was so advanced that no R0 resection was possible (20). Granowski-LeCornu et al. showed a 6-fold increased risk of death with distant metastases (25).

EOS patients also show a clear negative impact on survival in the presence of metastases. Hung et al. retrospectively analyzed the results of 202 patients with primary “high-grade” osteosarcomas (26). 22.3% of the patients showed metastases at the time of diagnosis (57.8% in the lung, 28.9% bone metastases, 11.1% showed metastases in both lung and bone). 5-year survival rate of this patient population, was 77.3% without metastases vs. 33.9% with metastases (26). Berner et al. analyzed 424 patients with extracranial osteosarcomas. Patients without metastases showed a 10-year survival rate of 48% vs. 11% with metastases (27). Hagleitner et al. also showed that the occurrence of metastases is associated with a poorer survival rate (23). Hung et al. compared the 5-year survival rate of 58 EOS patients without metastases (90.4%) and the survival rate of 16 patients with metastases (25%) (19).

### Survival rates in relation to resection margins in COS

**Table 3** shows the impact of R0 and R1 resection, respectively, on survival rates of patients with COS. Looking at the data in **Table 3**, the importance of free resection margins is quite clear. Survival rates in all studies considered decreased significantly when no free resection margins were achieved. Despite the consideration of many prognostic factors (age, gender, site, histological variant tumor size, presence of soft tissue extension, surgical margin positivity, neo-adjuvant chemotherapy, postoperative adjuvant treatment) the resection margin was the only factor influencing the survival rate in the study by Krishnamurthy et al. (8). Chen et al. (2017) were also able to demonstrate the importance of R0 resection in their results. 7 patients of the collective of Chen et al. (2017) could not be resected R0. The 5-year survival rate of these patients was 0% (15). Also, Thariat et al. showed that large-area resection and clear margins were the strongest prognostic factors for survival (28). Clean resection margins were shown to be the most important factor in preventing local recurrence. In patients with clear margins, 70.2% were recurrence free, with R1 resection only 38.5% (28). Thariat et al. concluded that resection with primary intent of free flap defect closure is the best method to achieve wide margins (28). The role of surgery is underlined by the finding that the median survival time of non-operated patients was only 6 months (28). Baumhoer et al. also showed the importance of free resection margins. Baumhoer et al. differentiated more precisely whether R0 resection of the primary tumor was performed or whether a recurrence was R0 resected or no R0 resection was achieved (20). If no recurrence developed in the patients and the primary tumor could be R0 resected, the 5-year survival rate was 92.3% (20). If one or more recurrence(s) developed and they could be completely resected subsequently the 5-year survival rate was 76.2% (20). If one or more recurrence(s) developed and they could not be completely resected subsequently the 5-year survival rate was 31.2%. If the R0 situation was not reached even after several surgical interventions (never R0), the 5-year survival rate was 21.8%, after 10 years it was 0% (20).

An interesting result regarding the resection margins was shown in the study of DeAngelis et al. If only narrow resection margins were achieved, this did not affect the survival rate. Positive resection margins, however, significantly reduced survival (7). Granowski-LeCornu et al. also examined several variables that affected the survival of patients with osteosarcoma of the jaws (25). Clear resection margins showed a significant impact on survival. Granowski-LeCornu et al. went into more detail about the width of the resection margins. It was shown that every 1 cm in resection margin width is associated with a 70% increase in survival (25). In contrast to De Angelis et al., Granowski-LeCornu et al. showed an association between metric R0 distance and survival (25). These contrasting results will be discussed later.

Seng et al. came to a similar conclusion as Granowski-LeCornu et al. when considering 55 patients with mandibular osteosarcomas. In the multivariate analysis, only surgical extension was found to be a significant factor for recurrence and death rate (29). Compared with non-survivors, survivors showed significantly wider resection margins (29). Resection far into healthy tissue, as achieved by hemimandibulectomy, significantly improves patient prognosis. The 5-year disease-specific survival rate was 23% for patients with partial mandibular resection and 89% for patients with hemimandibulectomy (29).

### Impact of therapeutic intervention on survival rates in patients with COS

Study results show a controversial picture regarding the best therapeutic option for COS. Due to the rarity of this tumor and the usually very small patient population, individual studies are difficult to compare. **Table 4** shows the data analyzed in this review regarding survival rates of COS patients according to therapy. A very important work, due to the representative patient collective and the good study design, is the study by Baumhoer et al.

Baumhoer et al. observed a comparatively large patient collective of 214 patients with osteosarcoma of the jaw and critically examines the therapy (20). Their patient collective was typical for COS patients. The median age of the patient population was 37 years and the tumors were primary osteosarcomas (20). The study provides survival data on different therapy options (surgery alone as well as neoadjuvant and/or adjuvant therapy). The aim of the study was to answer the question if multimodal therapy improves survival of patients with osteosarcoma of the jaw (20). Baumhoer et al. found no survival benefit by chemotherapeutic intervention in either the neoadjuvant or adjuvant setting, nor in a combination of neoadjuvant and adjuvant chemotherapy. On the contrary, patients who received chemotherapy showed worse survival rates than the comparison group without chemotherapeutic intervention (See **Table 4**) (20). The explanation of these results is that COS do not show shrinkage by chemotherapy due to the bone scaffold formed by the tumor cells (20). For this reason, Baumhoer et al. saw no indication for neoadjuvant chemotherapy in osteosarcomas of the jaw bones, since the tumor does not shrink and thus there is no improvement in resectability (only 2 good responders (< 10% vital tumor residues) and 6 bad responders (>90% vital tumor residues)) (20). Even in patients with local recurrence, chemotherapy showed no advantage in survival rates (20).

In contrast, Jasnau et al. showed a slight improvement in survival in patients treated with chemotherapy (see **Table 4**). However, the patient population here does not represent the classic COS patient. 49 patients were included in the study (27 maxillary and 22 extragnathic). The median age of the patients was 19.7 years and 27% of the tumors were secondary malignancies (30). The majority of these occurred in the area of an area previously treated by irradiation (30). All 49 patients received surgical therapy, 37 patients received chemotherapy, and 7 patients received chemotherapy and radiotherapy (no survival data are available for these 7 patients). Of the 44 patients who received chemotherapy, 23 received chemotherapy as adjuvant therapy and 21 received neoadjuvant chemotherapy (30). Jasnau et al. did not differentiate between neoadjuvant and adjuvant chemotherapy when reporting survival rates. Only survival with or without chemotherapy was reported. However, the conclusion of Jasnau et al, despite the slight survival advantage with chemotherapeutic intervention, is as follows: The localization in the jaw bone as well as the surgical R0 resection were shown to be significant factors for the survival of the patients (30).

Mücke et al. addressed the question of whether neoadjuvant chemotherapy improves outcome in adult patients with primary COS or not (31). Neoadjuvant chemotherapy provided a survival benefit in the analysis of Mücke et al. (31). The study included 36 patients (10 maxilla/26 mandible). 24 patients in the control group received surgical therapy, and 12 patients in the study group received neoadjuvant chemotherapy followed by surgical resection. Patients in the study group showed a better 5-year survival rate of 66.7% vs. 41,7% without neoadjuvant CT (31). The parameters influencing survival were tumor size, tumor location, neoadjuvant chemotherapy, and age. Mücke et al. came to the conclusion that early tumor detection, neoadjuvant chemotherapy and radical surgery are the most important factors for patient survival (31). However, the authors did not specify how patients are divided into study and control groups.

Chen et al. (2017) analyzed the prognostic and therapeutic parameters of 157 patients with COS (15). Looking at the disease-specific-survival, the use of chemotherapy did produce a short-term survival benefit, but this leveled off by year 2 after initial treatment (See **Table 4**). Looking at the 1- and 2-year disease-specific-survival, the best results were achieved by multimodality therapy consisting of surgery, chemotherapy, and radiotherapy (15). Patients treated with chemotherapy or radiotherapy and surgery, the survival advantage could only be shown for the first 2 years, after which it became relative (see **Table 4**). Patients who received multimodality therapy consisting of surgery, chemotherapy, and radiotherapy showed a minimal survival benefit related to disease-specific-survival for 4 years (15). When the 5-year disease-specific-survival and 5-year overall-survival of the patients were considered, the best survival rate was shown with purely surgical intervention (15).

Also very important is the study by Shim et al. Shim et al. analyzed data from 821 patients with COS from the National Cancer Database (NCDB) and compared 9 treatment cohorts with each other (surgery only, neoadjuvant chemotherapy + surgery, surgery+ adjuvant chemotherapy, neoadjuvant chemotherapy + surgery + adjuvant chemotherapy, surgery + adjuvant radiotherapy, surgery+ chemoradiation, chemoradiation, radiotherapy, and no treatment) (32). Shim et al. failed to show an overall survival benefit with chemotherapy (neoadjuvant or adjuvant), radiotherapy, or a combination of these therapies (32).

Interestingly, patients treated with neoadjuvant chemotherapy + surgery + adjuvant chemotherapy showed a survival advantage in the first 18 months compared to patients treated with surgery alone (95.8% vs. 78.5%). However, no long-term survival benefit could be achieved with chemotherapy or radiotherapy (32).

The aim of Thariat et al. was to find out the influence of the different treatment options on COS prognosis. Administration of neoadjuvant chemotherapy improved disease-free and metastasis-free survival in the analysis of Thariat et al. (28). It is a multicenter study of 111 patients with a median age of 35 years and a median tumor size of 4.5cm (range from 1.5-13 cm). 93.1% of patients received neoadjuvant chemotherapy. 54.7% adjuvant chemotherapy and 23.8 postoperative radiation (13 patients due to positive resection margins) (28). Patient survival correlated significantly with age, tumor size, and success of surgical resection (28). Thariat et al. does not provide precise data on how survival rates differed according to therapeutic intervention. The conclusion of Thariat et al. is that free flap surgery with wide margins is the treatment method of choice for COS (28).

Fernandes et al. examined 16 patients with osteosarcoma of the jaw bones. All patients received surgical therapy first (33). 4 patients received adjuvant chemotherapy. The patients who received chemotherapy were still alive at the final observation period. Of the 4 patients who did not receive chemotherapy, 2 died, indicating a trend for better survival with chemotherapy, but not statistically significant (33).

The study by Guo et al. deals with osteosarcomas localized in the skull base (18). In case of tumor localization in the skull base, a better survival rate was shown by radical surgery and the addition of chemo- and radiotherapy. The median survival time of patients treated only surgically was 18 months. Patients who received surgery and postoperative chemotherapy and radiotherapy survived an average of 50 months (18).

## Discussion

### Clinical behavior and prognosis of COS compared to EOS

In this paper, the differences in tumor biology shown in the literature, as well as the results of the different therapy concepts in EOS and COS should be shown and discussed. Looking at the results of this literature review, it appears that there are clinical differences in osteosarcomas with respect to their localization.

EOS occur at an earlier age and mainly affect adolescents and young adults (2). The 5-year survival rates of EOS are worse than those of COS. In the papers found in this literature search, the survival rate was approximately 50% for EOS. EOS are more frequent than COS and show a higher rate of metastasis. The presence of metastases and histologic response to chemotherapy significantly correlated with survival in EOS (25% vs. 90.4%) [55]. This also explains the current gold standard of therapeutic intervention in EOS. The therapy to be favored for OS of the long bones and axial skeleton is neoadjuvant chemotherapy, followed by surgical resection of the primary tumor and adjuvant chemotherapy. Mainly chemotherapy has improved the 5-year survival rate of EOS to 60-70% (6). Significantly worse survival data were observed when patients did not receive multimodal therapy (20). As mentioned in the introduction, EOS must be considered a systematic disease and 90% of patients develop metastases to the lungs when treated only surgically (20). This explains the poor survival rates without multimodal treatment.

If the resectability of a tumor is considered as a prognostically important factor, the extracranial osteosarcomas of the long tubular bones should show the best outcome, since here the resection can be best achieved relatively easily in healthy tissue. The fact that extracranial osteosarcomas of the long bones show a worse outcome (see **Table 2**) suggests that other factors also influence the survival rate. It is also an indication that craniofacial and extracranial osteosarcomas differ significantly biologically. In comparison to EOS it is shown that COS have a lower metastatic rate of 17,6% over a 5-year observation period (20). COS occur in the third to fourth decade of life (7, 10). Because of the proximity to anatomically complex and important structures and because of the aesthetic and functional demands in the facial region, the extensive surgical resection of COS is very complex (8, 10).

### Role of surgical resection and free margins

The aim of this review was to determine, based on the existing literature, the importance of surgical intervention and to what extent free resection margins influence the survival of patients with COS. When looking at the available studies, the importance of R0 resection is clearly shown. All studies analyzed in the present review show a survival benefit with R0 resection. No matter what patient population was considered in each study (patient age, radiotherapy-related second tumor, or tumor location of COS), the significance of R0 resection was always clear. The importance of R0 resection is outlined by the data presented in **Table 3**. Several studies (28, 30) show a clear survival benefit for R0. Also, Thariat et al. could show that large-area resection and clear margins were the strongest prognostic factors for survival (28). Clean resection margins were shown to be the most important factor in preventing local recurrence. Krishnamurthy et al., Jeong et al. and Baumhoer et al. even concluded that the R0 status is the only therapeutic parameter with influence on survival (8, 20, 34).

Chen et al. (2016) also highlighted the importance of R0 resection. Considering only the disease-free survival rate, R0 resection showed to be the only significant factor for survival (16).

Thus, the study evidence clearly shows the importance of R0 resection for the survival of patients with COS. However, it is unclear what the authors mean by wide margins. In most studies, they do not specify how far resection margins are in healthy tissue and what is the minimal distance to be labeled as “wide margin”. The studies analyzed here came to conflicting conclusions regarding the importance of the width of the resection margins. Granowski-LeCornu et al. and Seng et al. demonstrated an improvement in survival related to the metric width of the resection margin (25, 29). De Angelis, however, concluded that only free resection margins are important, but the width of the margins has no positive influence (7).

These different results are probably due to the accuracy of the histological preparation of the specimens. It must be noted that not every tumor region can be cut and assessed completely in pathology. Furthermore, it must be assumed that pathologic examination is not performed with equal techniques at every department. For this reason, there is a possibility that the tumor extension at close margins may still continue at one location and remain uncovered. It can therefore be assumed that the further the resection is carried out in healthy tissue, the lower the probability that a tumor cell will make contact with the resection margin.

### Role of tumor localization of COS

Furthermore, the prognostic influence of tumor localization in COS should be discussed.

The literature considered in this review shows a clear result regarding localization of COS and prognosis. Jasnau et al. concluded that patients with osteosarcomas of the jaw bones showed a significantly better 5-year survival rate of 88.1%. If the COS showed tumor localization in the skull base, the 5-year survival rate was 58.4% (30). The assumption of a better survival rate for localization of osteosarcomas in the jaw and the slightly worse survival rate in studies that consider craniofacial osteosarcomas in general can be seen in the following study. Guo et al. analyzed patients with osteosarcoma of the skull base. The 5-year survival rate of these patients was only 30.5% (18). Guo et al. explained the low survival rate by a high recurrence rate (1-year recurrence rate 47.0%, 2-year recurrence rate 68.8%) and proximity to complex and anatomically important structures (18). Mücke et al. also concluded that tumor location in the mandible influences outcome positively (31).

The best survival rates are found with localization in the mandible. Chen et al. (2016) showed significant survival advantage with tumor localization in the mandible (70% vs. 50.96%) (16). The conclusion of Chen et al. (2016) was that the only significant effect on the 5-year survival rate was the surgical intervention as initial therapy, the R0 resection and the localization of the tumor in the mandible (16). Chen et al. (2016) could not achieve tumor resection in healthy tissue in 7 patients. Considering the tumor location, in all 7 patients with R1 resection the primary tumor was located in the maxilla or the skull base. All 7 patients died within 5 years (16). Regarding metastasis, Chen et al. (2016) found out that patients who initially did not receive surgery and patients with tumor localization in the maxilla/skull base showed metastases more frequently (16). Meazza et al. reported similar results. Osteosarcomas located in the mandible had a better prognosis than those of the maxilla (14).

In a large-scale study by Lee et al. patients showed a longer median survival (10.4 years) of mandibular osteosarcoma compared to the rest of the skull/facial bone (6.3 years) (17).

It should be noted here that Lee et al. only differentiates between mandibula and remaining skull bone for tumor localization. Tumor localization in the maxilla is not considered separately.

The results of the literature considered show a clear survival advantage with tumor localization in the jaw bones. The best survival rates are found with localization of the osteosarcoma in the mandible. If one refers to the importance of R0 resection shown earlier in the text, the results now discussed are conclusive. Especially in the head and neck region an extended tumor resection is often difficult, because many important anatomical structures are located here (8). It must also be considered that in this region it is especially important to achieve an esthetically and functionally good surgical result (10). If the tumor extends far into the skull base, it is very difficult to achieve resection margins far into the healthy tissue. If the tumor is located in the mandible, R0 resection is easier to achieve (10). The easier resectability in the mandible explains the better prognosis when localization is in the mandible.

### Role of (neo)adjuvant chemo and radiotherapy

In contrast to EOS, there is no gold standard in therapeutic intervention for COS. The role of chemotherapy or radiotherapy is unclear. The individual authors in this review come to controversial conclusions regarding the benefit of chemotherapy (both neoadjuvant and adjuvant) as well as radiotherapy. In the literature search, only two studies demonstrated a 5-year survival benefit with neoadjuvant chemotherapy.

Mücke et al. were able to achieve an improved outcome with neoadjuvant chemotherapy in adult patients with primary COS (66.7% vs. 41.7%) (31). Thariat et al. also demonstrated a survival benefit with neoadjuvant chemotherapy (28). Thariat et al. only report a survival benefit with neoadjuvant chemotherapy; they do not provide precise data on how 5-year survival rates differ in percentage by therapy (28). However, the data of Thariat et al. must be viewed critically because 93.1% of the patients received neoadjuvant chemotherapy and the comparison group without chemotherapy is very small (28). The conclusion of the study is that free flap surgery with wide margins is the treatment method of choice for COS (28).

The other studies discussed now also show a survival benefit from chemotherapeutic intervention or multimodal therapy, based on a specific patient population. The results of these studies are therefore only valid for the specific patient population and it is very critical to question whether these results are generally transferable to patients with COS. The studies by Jasnau et al. and König et al. each deal with a very specific patient population (30, 35). In the patient collective of Jasnau et al. 27% showed osteosarcoma as a secondary tumor. The localization of the osteosarcomas was in already irradiated regions and the osteosarcomas must thus be seen as irradiation-induced. Furthermore, Jasnau et al. had a very young patient population (mean age 19.7 years) (30). In König et al, chemotherapeutic intervention was applied in the neoadjuvant setting (35). In the study by König et al. 50% of the patients have a previous malignancy or a familial predisposition. Neoadjuvant chemotherapy followed by adequate surgery showed a better survival rate than surgical intervention alone (35).

Both Jasnau et al. and König et al. were able to show a slight survival advantage by the use of neoadjuvant chemotherapy in their specific patient population (30, 35). In the study of Jasnau et al, chemotherapy was applied both neoadjuvantly and adjuvantly (30). In König et al, chemotherapeutic intervention was applied in the neoadjuvant setting (35). In the results of the two studies, it must be noted that patients with primary sarcomas had a significantly higher survival rate than those with secondary (radiation-induced) tumors (25).

Guo et al. considered osteosarcomas with localization of the skull base (18). In case of tumor localization in the skull base, a better survival rate was shown by radical surgery and the addition of adjuvant chemo- and radiotherapy. The median survival time of patients treated only surgically was 18 months. Patients who received surgery and postoperative chemotherapy and radiotherapy survived an average of 50 months (18). This suggests that for osteosarcomas of the skull base that cannot be resected in toto, a multimodality therapeutic approach of chemotherapy and radiotherapy potentially can provide a survival benefit.

The literature analyzed in this review contains two studies that were able to achieve a short survival benefit with multimodal therapy (15, 32). Chen et al. (2017) analyzed the survival rates of COS patients according to therapeutic intervention (15). Looking at the results of Chen et al. (2017) in relation to disease specific survival, there is a clear survival benefit at 1 year with chemotherapy or radiotherapy as well as a combination of the two (see **Table 4**) (15). When the 5-year disease specific and 5-year overall survival of the patients were considered, the best survival rate was shown with purely surgical intervention (15). Chen et al. (2017) already stated in the title that chemotherapy improves the survival rate of patients with COS. However, a detailed analysis of the data shows that the survival advantage of chemotherapy is only effective in the 1st year (15). Looking at the 5-year survival rate of patients by Chen at al.(2017) the positive effects of multimodal therapy leveled off again (15). Shim et al. showed a short-term survival benefit (18 months) of patients receiving both neoadjuvant and adjuvant chemotherapy. However, no long-term survival benefit could be obtained from multimodality therapy compared to surgery alone (32). Shim et al. explain this as follows. Adjuvant chemotherapy is effective in preventing distant metastases, which are very common in EOS (44-49%) (32). COS, on the other hand, metastasize much less frequently. In Shim et al, only 3.8% of patients showed distant metastases (32). Because of the infrequent metastasis of COS, Shim et al. view the use of adjuvant chemotherapy here critically (32). Due to the strong cytostatic effect, it is plausible that the survival rate can be improved for a few months. However, this must be weighed against the high toxicity of chemotherapeutic agents and poorer quality of life of patients.

In terms of long-term survival, both studies failed to show an advantage of multimodal therapy compared with surgery (15, 32). However, this must be weighed against the high toxicity of chemotherapeutic agents and poorer quality of life of patients

Fernandes et al. were able to show a trend for better survival with adjuvant chemotherapy, but these data was not statistically significant (33).

The majority of studies in this review failed to show a survival benefit from multimodal therapy in patients with COS. Krishnamurthy et al., Chen et al. (2016), Jeong et al, DeAngelis et al. and Granowski-LeCornu et al. were unable to show a survival benefit from either neoadjuvant or adjuvant chemotherapy in their studies (7, 16, 25, 34)

Of particular importance is the work of Baumhoer et al. due to the large patient collective and the exact characterization of the patient collective. Baumhoer et al. considered a comparatively large patient population of 214 patients. This is the typical COS collective. The study is very transparent, provides a lot of information and has a good study design. Chemotherapy - neither neoadjuvant and/or adjuvant - showed no benefit for the prognosis of the patients (20). On the contrary, there was even a negative association related to the outcome of the patients (5 year survival rate: with neoadjuvant CT 50.1% without neoadjuvant CT 69.9%/with adjuvant CT 47.3% without adjuvant CT 74.6%/combined therapy neoadjuvant and adjuvant CT 44% without combined therapy 68.8%) (see **Table 4**) (20). Because COS do not show shrinkage with chemotherapy and thus cannot be resected better, and because metastasis rate in COS is very low, Baumhoer et al. concluded that the key to curing osteosarcoma of the jawbone is to achieve the surgical R0 situation to prevent metastasis (20). The majority of patients with osteosarcoma of the jawbone are curable by surgical R0 resection (20). Multimodal therapy should be critically questioned. Baumhoer et al. criticizes that craniofacial osteosarcomas are treated the same as extracranial osteosarcomas (20). With regard to the results of Baumhoer et al. that clearly showed no survival benefit by multimodal treatment (see **Table 4**) it must be discussed why this study is cited in the German AWMF guidelines to underline the conclusion to equate craniofacial osteosarcomas with extracranial ones and to apply the same therapeutic concept (36).

The German AWMF guideline recommends radical resection in sano in combination with pre- and postoperative chemotherapy for the treatment of craniofacial osteosarcomas (36). As a source for this conclusion 3 papers are mentioned, which were also analyzed in this Review (Baumhoer et al., Jasnau et al. and Thariat et al). The study of Baumhoer et al. comes to a contrary conclusion regarding the benefit of chemotherapy (20). Jasnau et al. does not consider the classical patient collective of COS, as described earlier (a large proportion of the patients considered showed secondary malignancies and the median age of these patients also does not reflect the classic craniofacial osteosarcoma patient) (30). Thariat et al. showed a survival advantage by the use of multimodal therapy, but this must also be critically questioned, since 93% of the patients received neoadjuvant chemotherapy and the comparison group of patients who received only surgical therapy is very small (28).

The AWMF guidelines do not consider COS as a separate tumor entity. They do mention that metastasis is much less frequent and local recurrence is a major complication, but still equate COS with EOS. The recommended therapy of the guideline for COS is as follows: An en bloc resection of the tumor should be performed in combination with pre- and postoperative chemotherapy. The guidelines cite the 3 studies channeled in this work as sources for this treatment recommendation (20, 28, 30). Larger studies are clearly needed that also take into account the different initial tumor parameters of patients (36).

Like the AWMF guidelines, the ESMO Clinical Practice Guideline for diagnosis, treatment and follow-up recommends the same treatment for COS and EOS (37). The ESMO treatment recommendation for osteosarcoma also consists of chemotherapy (both neoadjuvant and adjuvant) and surgery of wide resection margins (37). A recommendation for radiotherapy is given for patients with unresectable primary tumors or where surgery would be unacceptably morbid (37). ESMO mentions that COS metastasize less frequently and that the role of chemotherapy is unclear. Nevertheless, ESMO recommends treating COS the same as EOS. The ESMO therapy recommendation also states that low-grade OS should only be treated surgically, but high-grade OS should always be treated multimodally (37). This contradicts again with the results of Baumhoer et al. and the results of the current review. The patient collective of Baumhoer et al. showed 92.1% from high-grade tumors (20). Nevertheless, the best survival rates were achieved with purely surgical intervention (20).

### Consequence for the treatment algorithm of COS

All clinical treatment decisions should be made in an interdisciplinary tumor-board. The results of this review failed to show evidence that chemotherapy in both the neoadjuvant and adjuvant settings or radiotherapy confer a survival benefit in COS. COS that are well resectable surgically should be treated surgically only. If very extensive COS are seen that extend into the skull base and are surgically not or only marginally R0 resectable, initially a second opinion should always be obtained from another major surgical center. It should be re-evaluated whether resectability is truly not a given. If this is confirmed, an individual decision must be made whether the patient can benefit from multimodal therapy including adjuvant/neoadjuvant chemotherapy and adjuvant radiotherapy. **Figure 1** shows the treatment algorithm suggested by the authors of this review. Unresectable COS should be treated by primary radiochemotherapy. Margin positive COS should be submitted to secondary surgery to achieve positive margins. Even functionally and aesthetically impairing resections should be performed to achieve R0 resections. The use of adjuvant chemo- or radio-chemotherapy should be discussed in cases in which R0 resection was only achieved in the second surgical attempt. However, there is no clear evidence for this recommendation in the literature.

### Limitations of the study

Considering the limitations of this review, the main focus should be on the heterogeneity of the study landscape. Due to the small patient population, the different treatment protocols and the large variance in tumor localization and size, the individual studies are difficult to compare. In addition, craniofacial osteosarcomas represent a very rare clinical entity. Therefore, a high number of cases can only be achieved within the framework of a multicenter analysis. The different approaches to osteosarcoma therapy in the individual centers would make such a prospective multicenter study very difficult. A generally accepted treatment guideline for COS is missing and a gold standard in therapy is not clearly defined. Furthermore, the comparatively long-time horizon of the studies cited in this review should not be neglected. The inclusion criteria define a period of twenty years and thus literature from the year 2000 onwards was included in this analysis. This must be considered as a further limitation of this study.

It can be assumed that during this period surgical procedures, radio- and chemo-therapy regimes have changed. Thus, the comparability of newer and older studies may be limited.

A further limitation results from the lack of studies that directly compare craniofacial and extracranial osteosarcomas. This could be due to the different disciplines involved in the treatment of craniofacial and extracranial osteosarcoma.

The following questions cannot be answered sufficiently with the available data and should therefore be addressed in future research:

How should tumors with R1 resection in the initial surgical approach and secondary R0 resection be handled? Should adjuvant therapy be applied or is follow-up alone sufficient for these cases?

Current data indicated that some COS patients might benefit from neoadjuvant chemotherapy. Therefore, it would be helpful to identify molecular markers to identify these patients to apply neoadjuvant chemotherapy selectively to suitable patients.

## Conclusion

The present review reveals the great heterogeneity in the study landscape regarding craniofacial osteosarcoma. Studies with mostly a small patient cohort with different tumor size, localization and treatment modalities were reported, making comparison and interpretation of data difficult. Nevertheless, the following statements could be derived by systematic literature analysis: Craniofacial osteosarcomas show a better 5-year survival rate compared to extracranial osteosarcomas. This is particularly evident when craniofacial osteosarcomas are localized in the jaw bones. Tumor localization in the mandibula shows a better prognosis compared to the maxilla. However, the survival rate decreases significantly if the base of the skull is also affected. This can be explained by the extreme difficulty or impossibility of R0 resection in these localizations. In terms of therapy, the most important factor in craniofacial osteosarcoma is the surgical achievement of R0 resection. Craniofacial osteosarcomas show less metastasis than extracranial osteosarcomas, but a higher rate of local recurrence. This is probably due to the difficulty in achieving wide tumor-free resection margins in the anatomically complex region of the facial skull. In contrast, the response to neoadjuvant chemotherapy has been shown to be the most important prognostic factor in extracranial osteosarcomas. The role of neoadjuvant chemotherapy in craniofacial osteosarcoma is controversially discussed.

A clear survival benefit from neoadjuvant and adjuvant chemotherapy could not be shown. There is no evidence of a benefit resulting from adjuvant radiotherapy in R0 resected cases.

**Figure 2:**
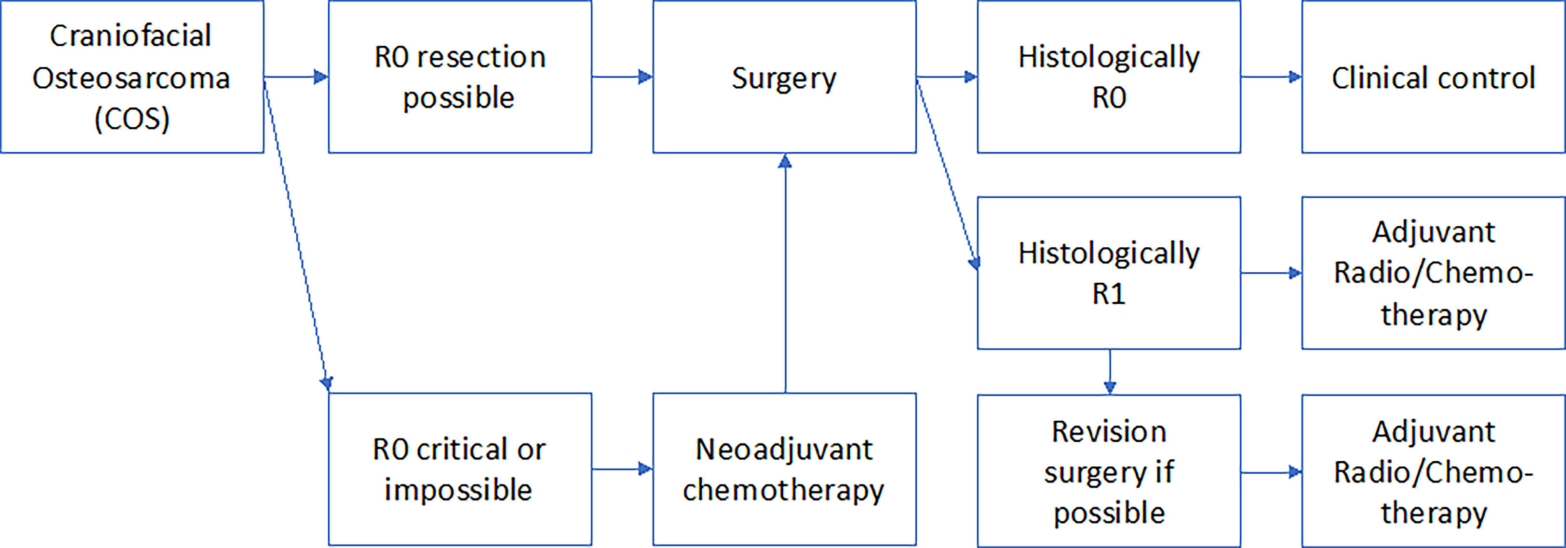
The Figure shows the authors’ proposed treatment strategy of COS related to the resection margins.

We recommend to treat most craniofacial osteosarcoma cases only surgically **Figure 2**. We advise the use of neoadjuvant chemotherapy in cases in which R0 resection is extremely unlikely due to the extension and localization of the primary tumor. We recommend adjuvant chemotherapy and radiotherapy when R0 resection was not successful in the initial surgical attempt. However final therapeutic decision should always be made in an interdisciplinary tumor-board.

The clinical and prognostic differences between craniofacial and extracranial osteosarcoma suggest that craniofacial osteosarcoma could be a biologically independent tumor entity that should be considered separately from extracranial osteosarcoma in studies.

## Data availability statement

The original contributions presented in the study are included in the article/**Supplementary Material**. Further inquiries can be directed to the corresponding author.

## Author contributions

VW conducted the literature analysis and wrote the manuscript. MW and RS contributed to the literature research and significantly contributed to the manuscript. RL and MK contributed to the manuscript. All authors contributed to the article and approved the submitted version.

## Supplementary material

Supplementary Table 1 shows the main search terms used and their connection search terms. The number of initially found literature is shown. It shows how many studies were screened out due to duplicates, by title and abstract, and how many studies were included in the work.

Supplementary Table 2 shows the exclusion criteria based on the PICO model. It shows how many studies are excluded because they deal with a different patient population, a different intervention or a different outcome.
