# Supplementary material for: Systematic review of craniofacial osteosarcoma regarding different clinical, therapeutic and prognostic parameters
Source: Front Oncol. 2023 Mar 24;13:1006622. doi: 10.3389/fonc.2023.1006622 (PMC10080080; doi:10.3389/fonc.2023.1006622)
Supplement: Supplementary Table 1 — shows the main search terms used and their connection search terms. The number of initially found literature is shown. It shows how many studies were screened out due to duplicates, by title and abstract, and how many studies were included in the work. [file Table_1.docx]

**Table S1**

AND

| **Synonyme** | **osteosarcoma** | **therapy** |  |  | Removal of duplicates | Results after title screening | Results after abstract screening | Included full texts |
| --- | --- | --- | --- | --- | --- | --- | --- | --- |
|  |  |  | **neoadjuvant chemotherapy** | 445 | 407 | 62 | 50 | 27 |
|  |  |  | **adjuvant chemotherapy** | 548 | 223 | 26 | 16 | 7 |
|  |  |  | **radiotherapy** | 786 | 416 | 16 | 8 | 6 |
|  |  |  | **surgical therapy** | 562 | 341 | 29 | 19 | 11 |
|  |  |  | **multimodal treatment** | 926 | 211 | 19 | 12 | 5 |
|  |  | **survival rate** | **stating** | 135 | 114 | 13 | 8 | 3 |
|  |  |  | **metastasis** | 546 | 299 | 22 | 15 | 6 |
|  |  |  | **local recurrence** | 203 | 108 | 26 | 17 | 6 |
|  |  |  | **tumor size** | 140 | 91 | 13 | 11 | 9 |
|  |  |  | **resection margins** | 40 | 30 | 3 | 3 | 2 |
|  | **osteosarcoma of the head and neck** | (2000) |  | 443 | 109 | 66 | 40 | 24 |
|  | **osteosarcoma of the jaw** | (2000) |  | 328 | 256 | 52 | 29 | 14 |
|  | **craniofacial osteosarcoma** | (2000) |  | 145 | 101 | 11 | 5 | 4 |

|  | **Aspect1** | **Aspect2** | **Aspect3** |
| --- | --- | --- | --- |

AND

**Table S1**

Table S1 shows the main search terms used and their connection search terms. The number of initially found literature is shown. It shows how many studies were screened out due to duplicates, by title and abstract, and how many studies were included in the work.

**Table S2**

| **Search terms** |
| --- |

| **Synonyme** | osteosarcoma | therapy |  | Results after removal of duplicates | Excluded because of PICO criteria (Title) | | |
| --- | --- | --- | --- | --- | --- | --- | --- |
|  |  |  |  |  | Patients | Intervention | Outcome |
|  |  |  | neoadjuvant chemotherapy | 407 | Ewing: 68  Others: 33  Case: 24  Animals: 6  In Vitro: 3 | 30 | 181 |
|  |  |  | adjuvant chemotherapy | 223 | Ewing: 40  Others: 30  Case: 31  Animals: 26  In Vitro: 5 | 14 | 51 |
|  |  |  | radiotherapy | 416 | Ewing: 112  Others: 66  Case: 33  Animals: 29  In Vitro: 9 | 32 | 119 |
|  |  |  | surgical therapy | 341 | Ewing: 58  Others: 65  Case: 29  Animals: 32  In Vitro: 5 | 21 | 102 |
|  |  |  | multimodal treatment | 211 | Ewing: 53  Others: 28  Case: 11  Animals: 6  In Vitro: 5 | 28 | 61 |
|  |  | survival rate | stating | 114 | Ewing: 23  Others: 11  Case: 0  Animals: 12  In Vitro: 4 | 8 | 43 |
|  |  |  | metastasis | 299 | Ewing: 27  Others: 38  Case: 2  Animals: 9 | 12 | 189 |
|  |  |  | local recurrence | 108 | Ewing: 13  Others: 23  Case: 5  Animals:0 | 10 | 31 |
|  |  |  | tumor size | 91 | Ewing: 14  Others: 12  Case: 1  Animals: 1  In Vitro: 0 | 6 | 44 |
|  |  |  | resection margins | 30 | Ewing: 9  Others: 6  Case: 0  Animals: 2  In Vitro: 0 | 1 | 9 |
|  | osteosarcoma of the head and neck | (2000) |  | 109 | Ewing: 6  Others: 4  Case: 17  Animals: 2  In Vitro: 0 | 2 | 12 |
|  | osteosarcoma of the jaw | (2000) |  | 256 | Ewing: 16  Others: 22  Case: 67  Animals: 15  In Vitro: 1 | 1 | 82 |
|  | craniofacial osteosarcoma | (2000) |  | 101 | Ewing: 10  Others: 11  Case: 30  Animals: 5  In Vitro: 0 | 3 | 31 |

**Table S2**

Table S2 shows the exclusion criteria based on the PICO model. It shows how many studies are excluded because they deal with a different patient population, a different intervention or a different outcome.
